# Relationship of Biochemical and Hematological Markers With Alcohol Withdrawal Severity

**DOI:** 10.7759/cureus.71914

**Published:** 2024-10-20

**Authors:** Amna Qureshi, Javeria Junaid, Niaz Shaikh, Ayesha Siddiqa, Arshee Khan

**Affiliations:** 1 Internal Medicine, Dubai Academic Health Corporation, Dubai, ARE

**Keywords:** alcohol biomarkers, alcohol withdrawal severity, clinical institute withdrawal assessment for alcohol‑revised, creatine phosphokinase (cpk), electrolyte imbalance

## Abstract

Background

Alcohol withdrawal severity is widely assessed and objectified using the Clinical Institute Withdrawal Assessment for Alcohol revised (CIWA-Ar) scale. However, the subjective nature of this scale has led to several studies that scrutinized the relationship of various blood parameters to assess withdrawal severity. The aim of this study was to analyze the relationship of various laboratory biomarkers with the severity of alcohol withdrawal.

Methods

This was a retrospective study of 200 cases admitted to Rashid Hospital, UAE, with the diagnosis of alcohol withdrawal syndrome. Severity was assessed using CIWA-Ar. The average CIWA-Ar score was calculated and analyzed against creatine phosphokinase (CPK) on admission days one, two, and three using a correlation coefficient. Sixteen other blood markers were also analyzed, including hemoglobin, mean corpuscular volume, white blood cell (WBC), platelets, alanine transaminase, aspartate aminotransferase, aspartate aminotransferase (AST)/alanine aminotransferase (ALT) ratio, gamma-glutamyl transferase, albumin, international normalized ratio, lactate dehydrogenase, sodium, potassium, magnesium, phosphate, and creatinine. Additionally, the duration of alcohol intake and the timing of the last alcohol intake were also examined in relation to the severity of alcohol withdrawal.

Results

Average CIWA-Ar exhibited a positive relationship with CPK on admission day one (r=0.2 (p=0.008)); day two (r= 0.25 (p=0.003)), and day three (r=0.42 (p<0.001)). WBC and AST showed a relatively rising trend with higher CIWA scores (average values, however, remain within the normal range). The results were not statistically significant (p>0.01). Serum potassium and magnesium levels followed a decreasing trend with rising CIWA (average values, however, remained within the normal range). These results were also not found to be statistically significant (p>0.1).

Conclusion

A high CPK level was associated with the severity of alcohol withdrawal syndrome (SAWS). Low serum potassium and magnesium and high WBC and AST can be associated with alcohol withdrawal severity. These routine laboratory tests can serve as objective indicators of severity in addition to using the CIWA-Ar score for AWS, enabling prompt management and prevention of complications.

## Introduction

Alcohol use disorder (alcohol abuse, alcohol dependence, alcohol addiction, and alcoholism) has been known to kill over three million people each year, accounting for up to 6% of global deaths and contributing to one in 20 deaths in the Southeast Asia Region as per a 2016 report by the World Health Organization [1,2]. The prevalence of alcohol use in the Middle East and North African countries in 2016 was estimated to be 593.0 per 100,000 population [3].

Alcohol acts on multiple neurotransmitters in the central nervous system, disturbing the fine balance between the excitatory and inhibitory pathways. Its two main actions include blocking the N-methyl-D-aspartate (NMDA) excitatory receptors and acting on the gamma-aminobutyric acid type A (GABA) receptors, which causes CNS depression [4]. Long-term use leads to upregulation of the excitatory NMDA receptors, which is why sudden abstinence causes neuronal and biochemical hyperactivity, leading to the characteristic symptoms of withdrawal syndrome such as sweating, tremors, hallucinations, headaches, disorientation, as well as seizures and delirium tremens in severe cases [5].

Alcohol withdrawal syndrome (AWS) can occur after sudden cessation of alcohol consumption, either after long-term regular drinking or an episode of binge drinking. Patients can develop delirium tremens (DT) within 24-72 hours, which is considered the most severe form of alcohol withdrawal syndrome [6]. The mortality in untreated cases of DTs has been reported to approach 5% to 15% or even 20% [7]. Severe alcohol withdrawal syndrome can prolong the hospital stay by an average of four days, and up to 70% of patients when admitted to the ICU may require mechanical ventilation [8]. Hence, predicting and diagnosing severe AWS early is important to avoid complications.

The Clinical Institute Withdrawal Assessment for Alcohol Scale (CIWA-Ar), a widely used clinical scoring system, categorizes the severity of alcohol withdrawal based on ten symptoms and signs, such as agitation, anxiety, auditory disturbances, clouding of the sensorium, headache, paroxysmal sweats, tactile disturbances, tremor, visual impairment, and vomiting.

Many studies have been performed to identify laboratory parameters associated with the severity of alcohol withdrawal. Patients with abnormal values of mean corpuscular volume (MCV), aspartate aminotransferase (AST), and high creatinine phosphokinase (CPK) activity were more likely to develop delirium tremens [9,10]. Lower levels of electrolytes, notably hypokalemia, are also postulated to contribute to withdrawal delirium [11]. Hypokalemia along with high homocysteine levels proved to have high sensitivity for the development of delirium tremens in patients who presented with withdrawal seizures [12]. Low platelet count was another biomarker associated with severe withdrawal [13]. A research study done in France for the same purpose showed that patients with a higher Cushman score >8 (a similar alcohol withdrawal severity score) had elevated ALT levels greater than 1.5 times the upper limit of normal [14]. Other predictive factors that have been extensively studied in the past include the amount of alcohol consumed before hospital admission and previous episodes of complicated withdrawal, which had a direct correlation with severity [15].

The most important element of the management of severe withdrawal is to prevent its development by identifying potential risk factors early on in the course of the disease. The CIWA-Ar scoring system is used to assess the severity of the established withdrawal syndrome but is unable to predict which patients are at risk of developing severe alcohol withdrawal [16]. No standardized risk prediction model has been formulated. Nor have any such studies been conducted in the Middle East. Identification of such predictive parameters can effectively lead to early recognition of alcohol withdrawal syndrome severity and improved patient outcomes.

This study aims to identify the severity of alcohol withdrawal through blood parameters that will help characterize patients into low-risk or high-risk, thereby guiding decisions about their timely management to reduce further complications and in-hospital morbidity and mortality.

## Materials and methods

Study design

This study is a retrospective observational analysis of patients admitted with alcohol withdrawal syndrome at Rashid Hospital, UAE, one of the largest tertiary care centers in Dubai from January 2020 to September 2021. The study involved gathering relevant data, including patients' medical histories as well as various hematological and biochemical markers, from the electronic medical system.

Setup and selection of participants

The investigation was carried out at Rashid Hospital, a prominent tertiary care facility in Dubai, United Arab Emirates. Renowned for its trauma and emergency care services, the hospital caters to a substantial patient population from both Dubai and the Northern Emirates.

Patients admitted through the emergency department during the study period with the diagnosis of alcohol withdrawal syndrome, as per the Diagnostic and Statistical Manual of Mental Disorders-5 (DSM-5) criteria, were included in the study. Patients with known comorbid medical conditions such as chronic liver disease, musculoskeletal trauma, myocardial injury, sepsis, endocrine, psychiatric illness, drug-induced liver injury, and muscle injury were excluded from the study.

Patients were classified into three groups based on the severity of alcohol withdrawal, as assessed using the Clinical Institute Withdrawal Assessment Alcohol Scale Revised (CIWA-Ar). Hospital clinical practice guidelines were employed to classify patients based on the severity of their withdrawal symptoms. A CIWA-Ar score of seven or lower indicated mild withdrawal, a score of eight to 14 indicated moderate withdrawal, and a score of 15 or higher indicated severe alcohol withdrawal symptoms, which pose an increased risk of seizures.

Lab parameters

A total of seventeen hematological and biochemical markers were investigated, including creatine phosphokinase (reference range: 0 - 167 U/L), hemoglobin (male: 13.8 - 17.2 g/dL; female: 12.1 - 15.1 g/dL), mean corpuscular volume (77.0 - 95.0 fL), white blood cell (3.6 - 11.0 10*3 /uL), platelets (150 - 410 10*3 / uL), alanine transaminase (0 - 41 U/L), aspartate aminotransferase (0 - 40 U/L), aspartate aminotransferase (AST)/alanine aminotransferase (ALT) ratio (<1.0), gamma-glutamyl transferase (GGT) (0 - 30 U/L), albumin (3.4 - 4.8 g/dL), international normalized ratio (0.8 - 1.1), lactate dehydrogenase ( 140 - 280 U/L), sodium (136 -145 mmol/L ), potassium (3.3 - 4.8 mmol/L ), magnesium (1.6 - 2.6 mg/dL), phosphate (2.7 - 4.5 mg/dL), and creatinine (0.70 - 1.20 mg/dL).

Outcome measures

Biochemical parameters were analyzed based on severity to assess the central distribution of parameters with the severity of alcohol withdrawal. Creatine phosphokinase (CPK) was examined on admission days one, two, and three to assess any relationship between CPK values and the severity of alcohol withdrawal. The duration of alcohol intake and the last intake of alcohol were also studied in relation to the severity of alcohol withdrawal.

Statistical analysis

Continuous variables were presented as mean ± standard deviation or median with interquartile range (Q1, Q3) as appropriate, and categorical data was organized into frequency and percentages. The correlation coefficient was used to study the association of CPK values with the severity of alcohol withdrawal. All tests were two-tailed, and the p-value was considered significant at <0.05. Data entry and statistical analysis were carried out with IBM SPSS Statistics for Windows, Version 20 (Released 2011; IBM Corp., Armonk, New York, United States). This study was approved by the Research and Ethical Approval Committee of Dubai Health Authority with reference number DSREC/RRP/2021/43.

## Results

During the study period, 245 patients were admitted with the diagnosis of alcohol withdrawal syndrome. The underlying co-morbid medical conditions led to the exclusion of 23 patients. An additional 22 patients were excluded since they had very low CIWA scores on presentation and had possible alternate diagnoses. Our study population included 200 patients, out of which only one was female. The majority (n=156, 78%) of the patients were of South Asian origin. Almost half of the patients (n=95, 47.5%) had seizures on admission (Table 1).

**Table 1:** Demography of patients and seizures on admission.

Demographics and patient characteristics	n (%)
Gender	
Male	199 (99)
Female	1 (1)
Nationality	
South Asia	156 (78)
Middle East	23 (11.5)
Africa	14 (7)
Other	7 (3.5)
Seizure on presentation	95 (47.5)

The CIWA score as recorded on days one, two, and three of hospital admission was noted down, and the average daily CIWA score was calculated for the convenience of analysis. As per the CIWA score, 69 patients (34.5%) had mild withdrawal, 78 (39%) had moderate withdrawal, and 53 patients (26.5%) had severe alcohol withdrawal (Table 2).

**Table 2:** Severity of alcohol withdrawal as per the CIWA-Ar scores. CIWA‑Ar: Clinical Institute Withdrawal Assessment for Alcohol‑revised

Alcohol withdrawal severity (CIWA score)	n (%)
Mild (<8)	69 (34.5)
Moderate (8-14)	78 (39)
Severe (>14)	53 (26.5)

Fifty patients presented within 24 hours, 75 within three days, and 52 within seven days of the last binge. Of those who presented early within 24 hours, the majority of them (19%) had a mild withdrawal on admission and during their hospitalization, while those who presented late, after three days, had moderate (37%), or severe withdrawal (36%). We also noticed that the severity of alcohol withdrawal was higher in those who had a total duration of alcohol intake of more than five years (47%) as opposed to those who had only been drinking for one year (4%) (Table 3).

**Table 3:** Alcohol-related variables. Descriptive analysis presented as mean (%). P<0.05 is considered statistically significant. * Data was missing for 23 subjects; ^ Data was missing for 73 subjects

Variables	Mild (%)	Moderate(%)	Severe(%)	P-value
Last alcohol intake*				
<12 hours	9 (13)	5 (6)	2 (4)	0.14
12-24 hours	13 (19)	11 (14)	10 (19)	0.68
1-3 days	27 (29)	29 (37)	19 (36)	0.93
4-7 days	14 (20)	21 (27)	17 (32)	0.32
Duration of alcohol intake^				
1 year	2 (3)	3(4)	2 (4)	0.94
2-5 years	14 (20)	8 (10)	6 (11)	0.18
>5 years	31 (45)	36 (46)	25 (47)	0.97

**Figure 1:**
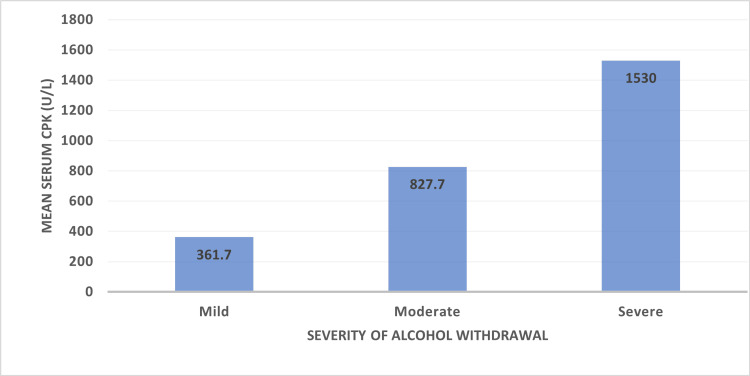
The X-axis reflects the severity of alcohol withdrawal syndrome as mild, moderate, and severe. The Y-axis reflects the mean serum CPK levels in relation to severity. CPK: creatine phosphokinase

The mean serum CPK levels were correlated to the severity of the withdrawal score. The mean CPK was 361.7 for mild, 827.7 for moderate, and 1530 for severe withdrawal scores (Figure 1). The CIWA scores and CPK levels were significantly correlated (P<0.05), indicating that patients with higher CIWA-Ar scores were likely to have higher serum CPK levels (Table 4).

**Table 4:** Correlation coefficient results showing the relation between average CIWA scores and CPK levels on days one, two, and three. P<0.05 is considered statistically significant. CIWA: Clinical Institute Withdrawal Assessment; CPK: creatine phosphokinase

	r coefficient	P-value
Day 1	0.2	0.008
Day 2	0.25	0.003
Day 3	0.42	<0.001

WBC also showed a rising trend with alcohol withdrawal severity. Average WBC count was 7.04 ± 2.9 in mild alcohol withdrawal, 7.71 ± 3.4 in moderate withdrawal, and 8.38 ± 3.9 in severe cases. However, the overall average values remained within the normal range, and the results were not found to be statistically significant (p=0.1). Similarly, AST levels were also found to rise proportionately with the severity of alcohol withdrawal. AST levels were 111 (69-228.5) in mild, 148 (80.75-265.5) in moderate, and 163 (81.5-243.5) in severe cases (p-value 0.45). The rise in ALT and GGT levels did not follow this pattern. GGT was 262 (137.25-699.5) in mild withdrawal, 403 (144-103.95) in moderate, and 372 (127.5-1184.5) in severe AWS (p=0.22). ALT levels were 64 (42-129) in mild, 84 (52-132) in moderate, and 71 (40-121) in severe withdrawal, but this was not found to be statistically significant p>0.1 (Table 5).

**Table 5:** Statistical comparison of biochemical and hematological parameters used in the study (n=200). Values are presented as mean ± standard deviation or median with interquartile range (Q1 – Q3). P<0.05 is considered statistically significant. WBC: white blood cell; ALT: alanine aminotransferase; AST: aspartate aminotransferase; GGT: gamma-glutamyl transferase; MCV: mean corpuscular volume; INR: international normalized ratio; LDH: lactate dehydrogenase; AWS: alcohol withdrawal syndrome

Parameters	Mild AWS	Moderate AWS	Severe AWS	P-value
WBC	7.04 ± 2.9	7.71 ± 3.4	8.38 ± 3.9	0.10
ALT	64 (42–129)	84 (52–132)	71 (40–121)	0.17
AST	111 (69–228.5)	148 (80.75–265.5)	163 (81.5–243.5)	0.45
GGT	262 (137.25–699.5)	403 (144–103.95)	372 (127.5–1184.5)	0.22
Potassium	3.83 ± 0.54	3.72 ± 0.58	3.68 ± 0.67	0.35
Magnesium	1.88 ± 0.42	1.79 ± 0.41	1.72 ± 0.51	0.37
Platelets	156 ± 72	144 ± 65	156 ± 70	0.40
MCV	93 ± 6.9	93 ± 7.2	95 ± 7.4	0.10
Creatinine	0.88 ± 0.37	0.96 ± 0.44	0.94 ± 0.3	0.45
Sodium	134 ± 3.95	133 ± 5.0	134 ± 5.3	0.03
Chloride	94 ± 4.9	92 ± 6.8	93 ± 7.2	0.21
Phosphate	2.8 ± 0.7	2.6 ± 1.2	3.08 ± 2.1	0.54
Albumin	4 (4–5)	4 (4–5)	4 (4–5)	0.59
AST/ALT Ratio	2 (1–2)	2 (1–2)	2 (1–3.5)	0.11
INR	1 (1–1)	1 (1–1)	1 (1–1)	0.27
LDH	289 (249–313)	330 (226–585)	280 (253–357)	0.78
Hemoglobin	14 (13–15)	14 (13–15)	14 (13–15)	0.97

A variation in electrolytes was seen among these patients. A significant decreasing trend was noted in magnesium and potassium levels with alcohol withdrawal severity (although values remained within normal limits). Potassium levels were 3.83 ± 0.54 in mild, 3.72 ± 0.58 in moderate, and 3.68 ± 0.67 in severe withdrawal (p=0.35). Magnesium levels were 1.88 ± 0.42 in mild, 1.79 ± 0.41 in moderate, and 1.72 ± 0.51 in severe cases (p=0.37). There was no correlation found between the average CIWA score and other blood parameters that included platelets, MCV, creatinine, sodium, chloride, phosphate, LDH, and hemoglobin, as shown in Table 5.

## Discussion

Alcohol withdrawal syndrome is a clinical syndrome associated with various hematological and biochemical abnormalities that may be related to the severity of the syndrome.

Creatinine phosphokinase (CPK), also known as creatinine kinase (CK), is an enzyme found in tissues that have a high energy requirement, mainly the brain, cardiac, and skeletal muscle, where different isoenzymes of the same exist. High CPK levels, specifically CK-MM, signify underlying skeletal muscle injury and are elevated in a variety of conditions like rhabdomyolysis, muscular dystrophy, myositis, burns, after strenuous exercise, etc. [17]. Alcohol is known to directly affect skeletal muscle, causing increased membrane permeability of muscles and allowing intracellular enzymes (including CPK) to leak out into the serum [18]. Alcoholic myopathy is characterized by weakness, pain, and muscle wasting [19]. Cessation of drinking has been shown to improve muscle function; however, persistent consumption will lead to further deterioration.

Muscle damage resulting from alcohol consumption is recognized as triggering rhabdomyolysis, characterized by elevated CPK levels, myalgia, and myoglobinuria [20]. This condition heightens the susceptibility to acute kidney injury. Individuals experiencing this complication may experience rapid deterioration, leading to substantial kidney damage and significant electrolyte imbalances, necessitating dialysis. Such interventions contribute to prolonged patient recovery time and can elevate inpatient morbidity and mortality rates [20]. Thus, an increase in CPK levels should be addressed immediately and treated with aggressive fluid repletion.

In this study, almost all the patients had high CPK on admission, in contrast to another study, which found high CPK in only 35% of patients, females more than males [21]. One possible explanation for the difference could be attributed to the different ethnic backgrounds, mainly Middle Eastern and South Asian subjects included in the current study.

Low serum sodium has been shown to cause a rise in CPK due to hyponatremia-induced rhabdomyolysis [22]. However, this study did not find any correlation between high CPK and low sodium levels. On the other hand, there was a positive trend in CPK in relation to alcohol withdrawal severity (Table 4). These results corroborate the findings of a previous study that compared the CPK levels in patients with alcohol dependence, alcohol withdrawal, and delirium tremens, where the latter two groups were found to have high serum CPK activity, with the highest number recorded in the delirium tremens group [10].

As concluded in one study, CPK levels following cessation of heavy alcohol drinking showed a delay of 24-48 hours before it began to rise, reaching a peak in three to five days and then returning to normal within two weeks [23]. In the current study, CPK levels increased significantly during the first 48-72 hours of admission. The difference could be explained by the fact that the majority of patients (n=95) in the current study suffered seizures on admission, which might also contribute to the rise of their CPK levels.

Alcohol is considered to be a bone marrow suppressant, resulting in thrombocytopenia, leukopenia, and anemia [24]. In contrast, the current study population showed a normal range of leukocyte count. This study also showed a low platelet count in AWS. However, a progressive downward trend was not observed among the three groups. Earlier studies have shown that thrombocytopenia is associated with severe alcohol withdrawal syndrome [13,25]. The initial low platelet count is still important to assess before the start of treatment, as thrombocytopenia and subsequent rebound thrombocytosis are associated with the onset of brain infarction in alcoholics [26].

AST and GGT showed higher initial values on admission, with a rising trend with alcohol withdrawal severity. One explanation is that of a parallel phenomenon, i.e., they reflect the effects of long-term and heavy alcohol consumption since most of the patients in this study with SAWS had a longer duration of alcohol intake >five years. However, these results were not found to be statistically significant (p>0.1).

Both potassium and magnesium exhibit a positive downward trend with escalating severity, a correlation supported by additional studies [11]. Hypokalemia is commonly observed in alcohol withdrawal syndrome and is ascribed to multifactorial causes. During the withdrawal phase, elevated levels of catecholamines prompt a shift of potassium and magnesium into the cells. Two additional factors contributing to this phenomenon are increased kaliuresis due to concurrent hypomagnesemia and respiratory alkalosis [27]. The repercussions of hypokalemia and hypomagnesemia can be severe, leading to complications such as paralytic ileus, arrhythmias, seizures, and ultimately, cardiac arrest.

A higher ALT, AST/ALT ratio was seen in patients with alcohol withdrawal syndrome, but a progressive rising trend was not seen in this study. The higher values of these liver function tests in all groups of patients may also support parallel phenomena demonstrating markers of excessive long-term alcohol consumption.

Limitations

In this retrospective study, there were a few limitations due to missing data in electronic medical records, particularly regarding the exact amount and nature of alcohol consumed by the patients. Additionally, the initiation of treatment in the emergency department before admission may have impacted the development and progression of withdrawal symptoms, affecting patient categorization based on severity. Furthermore, accurate determination of CPK levels was hindered by the need to promptly initiate treatment upon presentation to the emergency department.

## Conclusions

Various hematological and biochemical abnormalities are associated with the clinical syndrome of alcohol withdrawal. A high CPK level is associated with severe alcohol withdrawal syndrome (SAWS). Routine laboratory tests can serve as objective indicators of the severity of the alcohol withdrawal syndrome, enabling a prompt and proactive treatment approach to mitigate complications such as rhabdomyolysis-related acute kidney injury and arrhythmias induced by hypokalemia. However, more research is needed with a larger sample size to prove a relationship with other parameters. Till then, CIWA-Ar remains a reliable tool to assess the severity of alcohol withdrawal.
